# Nd:YAG Laser Photodisruption for Multilevel Premacular Hemorrhage due to Isolated Retinal Venous Macroaneurysm

**DOI:** 10.1155/2017/4630187

**Published:** 2017-10-04

**Authors:** Kenan Sonmez, Pehmen Y. Ozcan

**Affiliations:** ^1^Ulucanlar Eye Training and Research Hospital, Ankara, Turkey; ^2^Sanliurfa Education and Research Hospital, Sanliurfa, Turkey

## Abstract

A 55-year-old man presented with sudden deterioration of vision in the right eye. His visual acuity was reduced to hand motion because of a large multilevel premacular hemorrhage. Nd:YAG laser was performed to drain the entrapped hemorrhage under the internal limiting membrane (ILM) and posterior hyaloid face in the macula into the vitreous. Immediately after laser treatment, streaming of red blood cells into the vitreous gel through the perforation site was observed. At the first-month follow-up, BCVA improved to 20/25 and ILM wrinkling was observed at the macula where the preretinal hemorrhage cleared. Fluorescein angiography revealed an isolated retinal venous macroaneurysm located on the macular branch of the superotemporal vein at the bifurcation site. In contrast to retinal arterial macroaneurysms, retinal venous macroaneurysms are quite rare. To the best of our knowledge, this is the first case reported with multilevel premacular hemorrhage caused by an isolated retinal venous macroaneurysm.

## 1. Introduction

Various retinal vascular and hematologic disorders can cause premacular hemorrhage leading to a sudden decrease in vision [1]. Preretinal hemorrhages usually occur at the interface between the posterior hyaloid and internal limiting membrane (ILM). Less frequently, they are located between the ILM and the retinal nerve fibre layer. Although sub‐ILM hemorrhages have been most commonly associated with Valsalva retinopathy and Terson's syndrome, retinal macroaneurysms may also cause sub-ILM hemorrhages [2].

Unlike retinal arterial macroaneurysms, retinal venous macroaneurysms are extremely rare and can be either isolated or associated with venous occlusive disease [3, 4]. After a thorough search of literature, very few cases with premacular hemorrhage due to retinal venous macroaneurysm have been reported [5, 6]. In this case report, we describe a patient who had a recent multilevel premacular hemorrhage caused by an isolated retinal venous macroaneurysm and was treated with a pulsed Nd:YAG laser to drain the entrapped blood into the vitreous. To the best of our knowledge, this is the first case reported with multilevel premacular hemorrhage caused by an isolated retinal venous macroaneurysm.

## 2. Case Report

**Figure 1:**
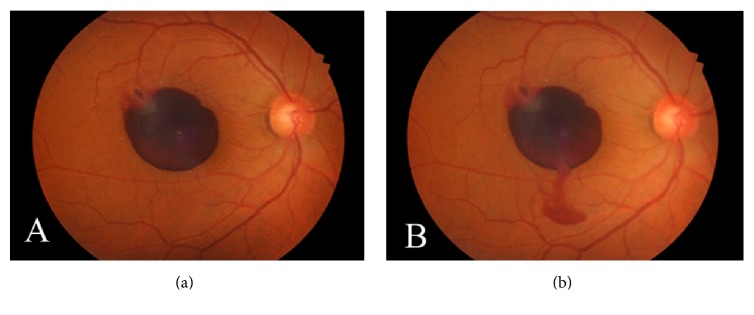
A 55-year-old man had sudden decreased visual acuity in the right eye. (a) Fundus photography of the right eye shows multilevel macular hemorrhage occupying nearly the half of macular area within the vascular arcade. Note the round dark hemorrhage which was considered to be a hemorrhagic detachment of ILM and a more superficially situated lighter-colored hemorrhage considered to be subhyaloid hemorrhage. (b) An immediate stream of blood into the vitreous through the photodisruption cleft is seen after the laser treatment.

**Figure 2:**
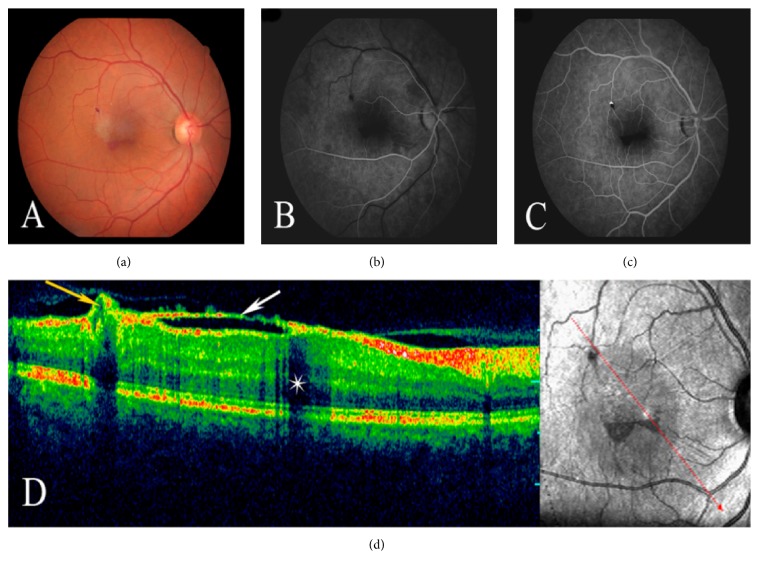
One month after laser treatment, (a) fundus photography of the right eye shows only small amount of residue blood at the lower edge of the detached ILM. (b, c) Fluorescein angiography shows a round area of hyperfluorescence which is prominent in venous phase and corresponds to a macroaneurysm of the macular branch of the superotemporal vein at the bifurcation site. (d) Optical coherence tomography demonstrates the detached and wrinkled ILM (white arrow) extending from the site of the macroaneurysm (yellow arrow) to the inferior margin of the residual sub-ILM blood causing a shadowing effect (star) in the macula.

A 55-year-old man presented with sudden visual loss in his right eye 4 days before he was examined in our retina clinic. He denied any history of trauma or ocular or systemic disease. His family history was unremarkable. The visual loss was not associated with a history of physical exertion. On examination, his best corrected visual acuity (BCVA) was hand motion in the right eye and 20/20 in the left eye. The ocular examination of his left eye was normal. Intraocular pressure was 12 mmHg bilaterally. Slit lamp biomicroscopy of the right eye revealed a normal anterior segment. Fundus examination showed a round dark hemorrhage which was considered to be a hemorrhagic detachment of the ILM and a more superficially situated lighter-colored hemorrhage considered to be subhyaloid hemorrhage, covering nearly half of the macular area within the vascular arcade (Figure 1(a)). Results of complete physical and laboratory evaluation were normal. The patient underwent Nd:YAG laser photodisruption of the posterior hyaloid and ILM. Laser exposures were started with low energies of 3 mJ and then gradually increased until perforation became visible at the surface of the hemorrhage. After Nd:YAG laser photodisruption was performed in the lower and most prominent area of the hemorrhage, and premacular hemorrhage immediately drained through the photodisruption cleft into the vitreous cavity (Figure 1(b)). No evidence of damage to the retina or choroid due to treatment was observed. BCVA rapidly improved to 20/40 within 2 weeks. At the first-month follow-up, BCVA in the right eye improved to 20/25 and only a small amount of residue blood was observed at the lower edge of the detached ILM (Figure 2(a)). Also, ILM wrinkling was observed at the macula where the preretinal hemorrhage cleared (Figure 2(a)). Fluorescein angiogram revealed a round area of hyperfluorescence corresponding to a macroaneurysm of the macular branch of the superotemporal vein at the bifurcation site (Figures 2(b) and 2(c)). No additional vascular or retinal abnormalities were found. Optical coherence tomography demonstrated detachment of the wrinkled ILM extending from the site of the macroaneurysm to the inferior margin of the residual sub-ILM blood (Figure 2(d)). This residual sub-ILM hemorrhage completely cleared at the fourth-month follow-up. Since follow-up examinations during the first year showed no changes and the patient did not complain of metamorphopsia and had good visual acuity, he refused further operations for the wrinkled ILM.

## 3. Discussion

Localized dilatation of the wall of a blood vessel is referred to as an aneurysm. Aneurysms arising on main retinal blood vessels within the first 3 orders of bifurcations are termed macroaneurysms and four distinct categories of macroaneurysms have been identified: arterial, capillary, collateral-associated, and venous [7]. In contrast to retinal arterial macroaneurysms, retinal venous macroaneurysms are quite rare and have almost exclusively been reported to develop after branch retinal vein occlusions [3, 7]. Very few cases of isolated retinal venous macroaneurysm without venous occlusion have been described in the literature [4–6, 8]. Since isolated retinal venous macroaneurysm is so rare, the course and complications of these lesions are not well-known. After a thorough Medline search, we could find few case reports, in which acute macular hemorrhage due to isolated retinal venous macroaneurysm was presented [5, 6]. In these reports, premacular hemorrhages were successfully treated with either Nd:YAG or argon green laser. However, the uniqueness of our case is the presence of acute multilevel premacular hemorrhage due to an isolated retinal venous macroaneurysm.

Proposed treatment options for premacular hemorrhage consist of observation, vitrectomy, and laser (either Nd:YAG or argon green laser) photodisruption. Although spontaneous clearing of premacular hemorrhage occurs, long-standing blood that is sequestered over the macula may increase the risk of epiretinal membrane (ERM) formation and toxic injury to the retina due to prolonged contact with hemoglobin and iron [9, 10]. In comparison to vitrectomy, the laser procedure is an ambulatory and painless procedure resulting in rapid visual rehabilitation. However, some rare complications related to laser procedures, including macular hole, parafoveal macular hole, retinal detachment, unsealed internal limiting membrane ERM formation, and persistent premacular cavity, have been reported [11–14]. Probably the formation of ERM is more common in cases of sub‐ILM hemorrhage, in which laser drainage requires disruption of the basal lamina of the sensory retina, with a consequent gliotic wound‐healing response. In the present case, Nd:YAG laser photodisruption of the posterior hyaloid and ILM was performed to drain the entrapped blood into the vitreous and visual acuity rapidly improved within 2 weeks. However, 1 month after the treatment, ILM wrinkling was observed as a complication after Nd:YAG laser treatment although the patient did not complain of visual impairment or metamorphopsia.

In conclusion, to the best of our knowledge, this is the first case reported with multilevel premacular hemorrhage caused by an isolated retinal venous macroaneurysm. Since this lesion is so rare, the subsequent course and complications of venous aneurysms are unknown. Nevertheless, isolated retinal venous macroaneurysm may be complicated by multilevel premacular hemorrhage and Nd:YAG laser drainage may yield very good functional results, with potential risk of ILM wrinkling or secondary membrane formation.
